# [Retracted] Methylene blue relieves the development of osteoarthritis by upregulating lncRNA MEG3

**DOI:** 10.3892/etm.2024.12562

**Published:** 2024-05-08

**Authors:** Xinyi Li, Chaoliang Tang, Jin Wang, Peipei Guo, Chengyao Wang, Yanlin Wang, Zongze Zhang, Huisheng Wu

Exp Ther Med 15:3856–3864, 2018; DOI: 10.3892/etm.2018.5918

Following the publication of this paper, it was drawn to the Editors’ attention by a concerned reader that the histological images shown in Fig. 1E on p. 3859 and the western blotting data shown in Fig. 4B on p. 3861 were strikingly similar to data appearing in different form in other articles written by different authors at different research institutes that had already been published elsewhere prior to the submission of this paper to *Experimental and Therapeutic Medicine*. In view of the fact that the abovementioned data had already apparently been published previously, the Editor of *Experimental and Therapeutic Medicine* has decided that this paper should be retracted from the Journal. After having been in contact with the authors, they accepted the decision to retract the paper. The Editor apologizes to the readership for any inconvenience caused.

